# Multiple cerebral infarctions after intravenous immunoglobulin for Guillain–Barré syndrome: two case reports and review of the literature

**DOI:** 10.3389/fimmu.2024.1433240

**Published:** 2024-07-23

**Authors:** Weisen Wang, Chunhua Feng, Yanqun Liu, Yi Tao, Xiaoying Bi, Xiaojun Hou

**Affiliations:** ^1^ Department of Neurology, the First Affiliated Hospital of Naval Medical University, Shanghai, China; ^2^ Department of Neurology, The 983rd Hospital of Joint Logistics Support Forces of the PLA, Tianjin, China

**Keywords:** Guillain-Barré syndrome, multiple cerebral infarctions, intravenous immunoglobulin, acute bulbar palsy, vascular risk factor

## Abstract

**Background:**

Guillain–Barré syndrome (GBS) is a polyradiculoneuropathy mediated by the immune system and is the primary reason for acute flaccid paralysis. Intravenous immunoglobulin (IVIg) is a recognized immunotherapeutic drug that can accelerate recovery from GBS. Limited literature exists concerning cerebral infarction complications with IVIg following its use in the treatment of GBS.

**Case presentation:**

A patient was diagnosed with the acute inflammatory demyelinating polyradiculoneuropathy subtype of GBS, while another patient was diagnosed with the acute bulbar palsy variant of GBS 2 years prior and experienced a relapse of GBS. Both patients received immunoglobulin therapy, during which multiple acute cerebral infarctions were detected using magnetic resonance imaging. Both patients had a history of coronary artery atherosclerotic heart disease and vertebral artery stenosis, and D-dimer and fibrinogen degradation products were significantly elevated after immunoglobulin therapy.

**Conclusions:**

The risk of cerebral infarction associated with IVIg is generally low in patients with different GBS variants. Nevertheless, the occurrence of cerebral infarction associated with IVIg might not be insignificant in older patients with vascular risk factors and should be carefully monitored.

## 1 Introduction

Guillain–Barré syndrome (GBS) is a rare but serious inflammatory disease that affects the peripheral nervous system (1). It has a worldwide annual incidence of about 1−2 cases per 100,000 person-years (1). GBS can lead to rapid-onset muscle weakness and paralysis, making it a potentially life-threatening condition (2). The acute bulbar palsy (ABP) variant of GBS can develop in the absence of prominent limb weakness in GBS, and the proportion of this GBS subtype is less than 1% (3). Although GBS is a monophasic disease, relapse can occur in 2–5% of patients (4).

Intravenous immunoglobulin (IVIg) is the first-line treatment for GBS (5). While IVIg products typically exhibit favorable safety profiles, examinations conducted in laboratories, instances detailed in case reports, and previous observational investigations have indicated a potential connection between IVIg products and heightened susceptibility to severe thromboembolic adverse events (TEEs) (6). There are fewer reported cases of cerebral infarction than of deep vein thrombosis and myocardial infarction (7). Here, we report two cases of multiple cerebral infarctions after IVIg treatment for GBS and review previous case reports.

## 2 Case presentation

### 2.1 Case 1

**Figure 1:**
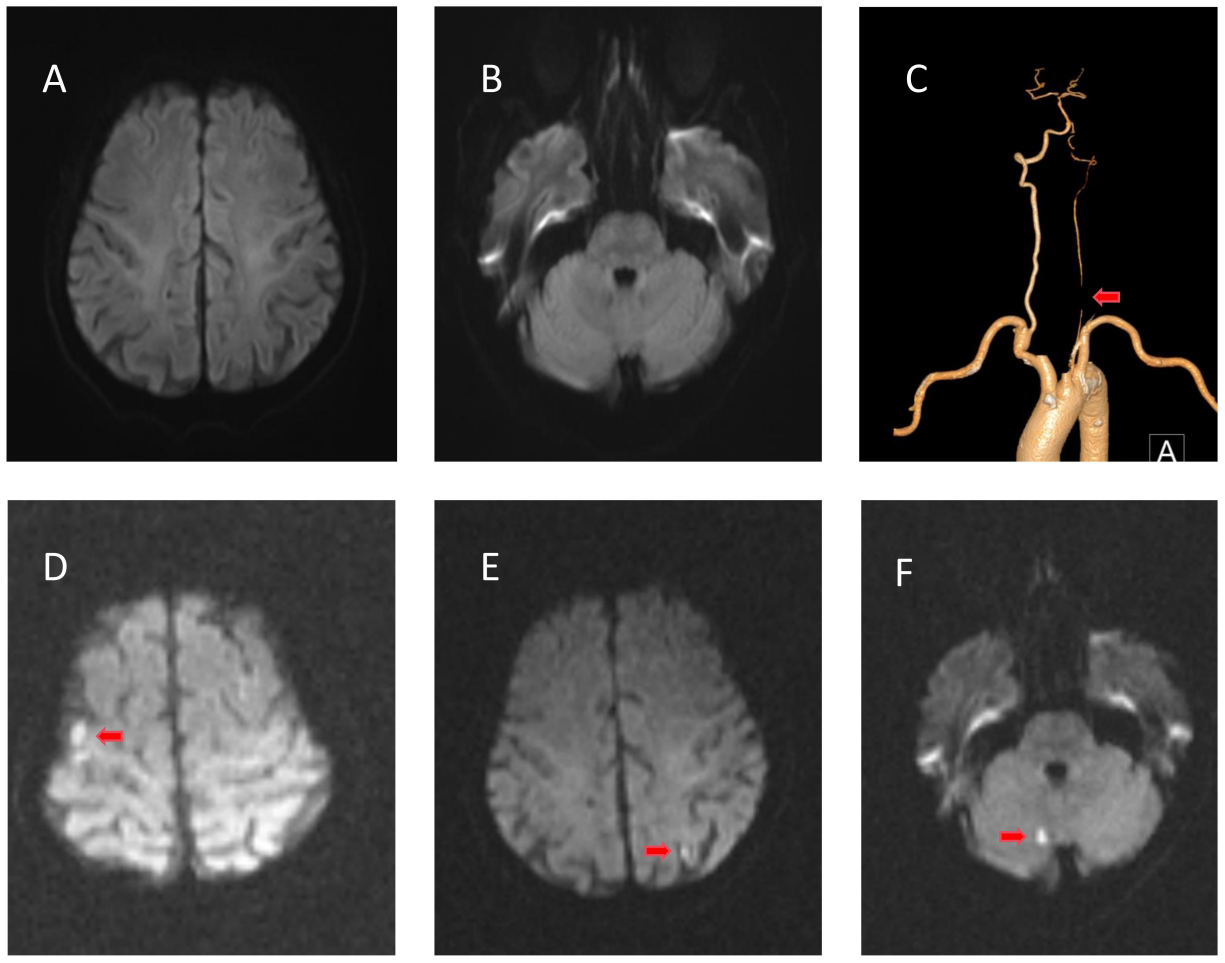
Radiological examinations of case 1 before and after intravenous immunoglobulin (IVIg). **(A, B)** No evidence of cerebral infarction before IVIg on diffusion-weighted imaging (DWI). **(C)** Computed tomography angiography showing occlusion of the intracranial segment of the left vertebral artery. **(D–F)** DWI of the right cerebellum, left frontotemporal cortical area, and right frontal cortical area showing restricted diffusion of the lesion after IVIg.

A 75-year-old man was hospitalized on May 20, 2023, following a 10-day history of weakness and abnormal sensation in the lower limbs and a 4-day history of dysarthria. Precisely 2 weeks prior, he had had a viral upper respiratory tract infection. He was unable to walk independently and exhibited symptoms of dysarthria, dysphagia, and dizziness. The patient had a history of coronary artery atherosclerotic disease for 12 years and hypertension for 10 years. The patient underwent coronary artery stent implantation and received oral clopidogrel treatment. There was no history of atrial fibrillation. He reported a history of smoking and alcohol consumption. Neurological examination on admission revealed bilateral peripheral facial paralysis, bilateral sluggish gag reflexes, and 4/5 on the Medical Research Council (MRC) scale for both lower limbs. Tendon reflexes of all four limbs were absent. Sensory examinations revealed a significant loss of pain and temperature sensation in both the hands and feet. There was no autonomic nervous system damage. Cerebrospinal fluid (CSF) analysis showed regular cell numbers and a raised protein level of 1050 mg/L on day 10 following the commencement of symptoms. Tests for immunoglobulin G (IgG) antibodies against GD1a, GD1b, GM1, GM1b, GalNAc-GD1a, GQ1b, and GT1a were negative. Excluding serum AQP4 antibodies that were positive at a titer of 1:10, the patient’s blood test results were otherwise normal. Nerve conduction studies revealed focal segmental demyelination. Computed tomography angiography (CTA) showed an occlusion of the intracranial segment of the left vertebral artery, with no significant abnormalities observed in the aortic arch (**Figure 1C**). Transesophageal echocardiography showed congenital bicuspid aortic valve deformity with moderate-to-severe regurgitation (7 mL), and patent foramen ovale was excluded. No arrhythmia was observed on cardiac monitoring. The patient did not exhibit lower limb edema during the illness, and lower limb vascular ultrasound excluded the possibility of deep vein thrombosis in the lower limbs. The patient was diagnosed with the acute inflammatory demyelinating polyradiculoneuropathy subtype of GBS. The patient had a low titer of AQP4 antibodies, but based on the clinical presentation and imaging findings, neuromyelitis optica spectrum disorders and other GBS subtypes such as Miller Fisher syndrome (MFS) were not considered. The patient began receiving immunoglobulin treatment at 0.4 g/kg/d for 5 days starting on May 21.

**Figure 2:**
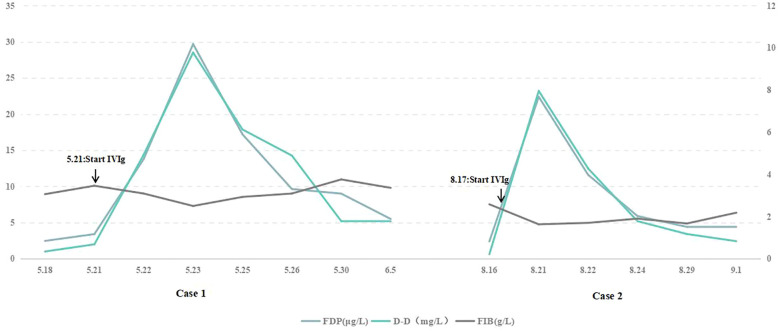
D-dimer and fibrinogen degradation products were progressively elevated after IVIg. Horizontal axis: date. Vertical axis on the left: fibrinogen degradation products. Vertical axis on the right: D-dimer, fibrinogen. D-dimer reference range: ≤0.55 mg/L; fibrinogen degradation product reference range: ≤5 μg/L. IVIg, intravenous immunoglobulin.

His muscle strength decreased on May 22 (3/5 MRC on the lower limbs and 4/5 on the upper limbs), and D-dimer (reference range: ≤0.55 mg/L) and fibrinogen degradation products (reference range: ≤5 μg/L) were progressively elevated (**Figure 2**, left). Cranial magnetic resonance imaging (MRI) revealed no evidence of cerebral infarction before the patient was admitted to our hospital (**Figures 1A, B**). On May 23, cranial MRI revealed multiple cerebral infarctions in the right cerebellum, left frontotemporal cortical area, and right frontal cortical area (**Figures 1D–F**). After the diagnosis of cerebral infarction, low-molecular-weight-heparin (LMWH) was added to the treatment plan. Upon discharge, the patient had bilateral peripheral facial paralysis and muscle strength in all limbs graded as 5. After 3 months, the facial paralysis had resolved, and his modified Rankin Scale (mRS) score was 1. The patient underwent transcatheter aortic valve replacement at another hospital.

### 2.2 Case 2

**Figure 3:**
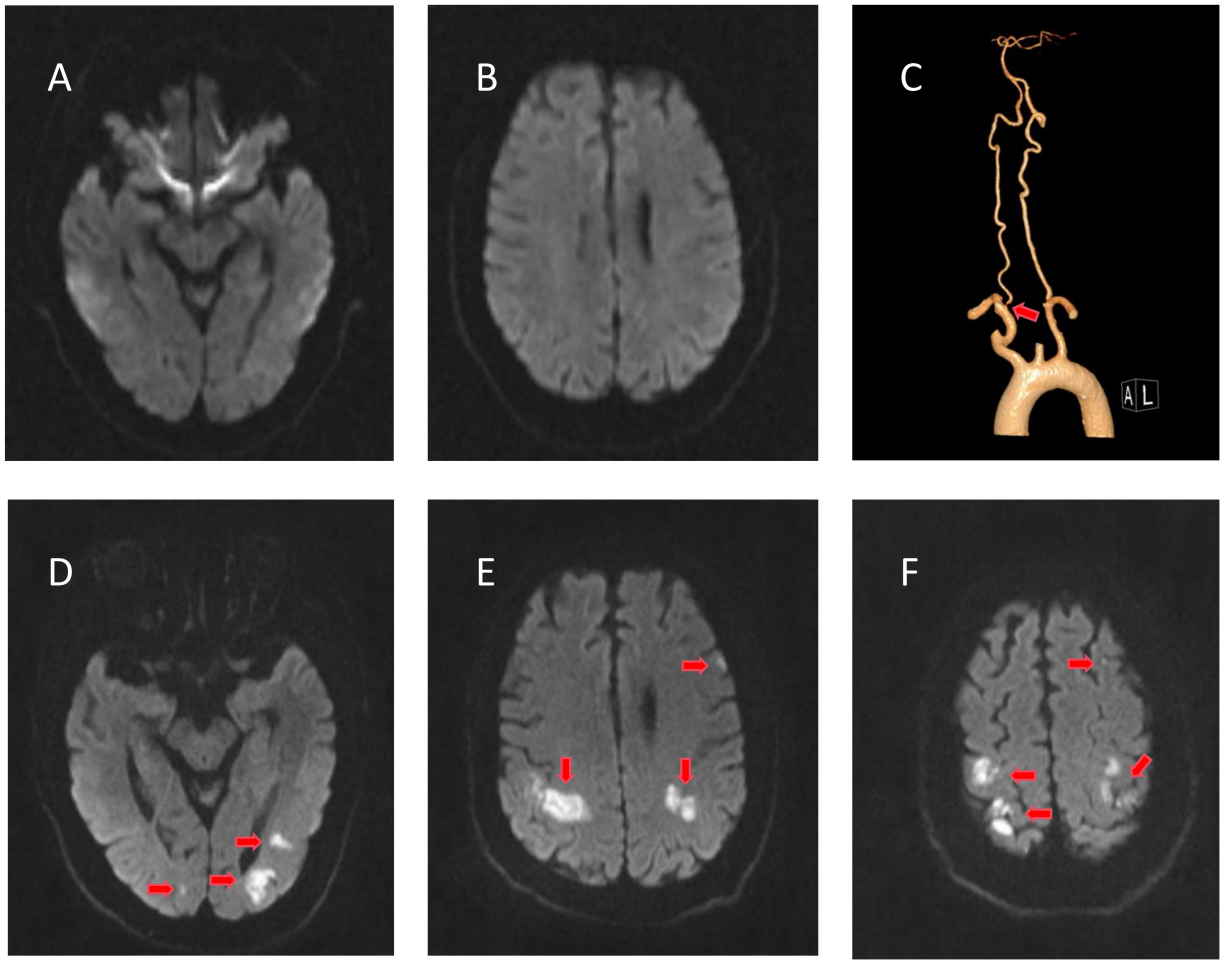
Radiological examinations of case 2 before and after intravenous immunoglobulin (IVIg). **(A, B)** No evidence of cerebral infarction before IVIg on diffusion-weighted imaging (DWI). **(C)** Computed tomography angiography showing right vertebral artery stenosis. **(D–F)** DWI of the bilateral parieto-occipital and left frontal lobes showing restricted diffusion of the lesion after IVIg.

A 65-year-old man was diagnosed with the ABP variant of GBS in August 2021 and presented with progressive dysphagia for 5 days. CSF analysis revealed normal cell counts and elevated protein levels (620 mg/L). IgG antibodies against GM2 were reported. After receiving IVIg, the dysphagia completely resolved. The patient was readmitted on August 15, 2023, with complaints of dysphagia. The patient had a 5-year history of coronary artery atherosclerotic disease and oral aspirin treatment. There was no history of atrial fibrillation. He also had a history of smoking. No other positive neurological signs were observed except for dysphagia. CSF analysis on day 5 revealed normal cell counts and an elevated protein concentration of 502 mg/L. IgG antibodies against GM2 and other antibodies yielded negative results. Results of laboratory examinations and nerve conduction tests were normal. Multiple electrocardiograms were conducted during hospitalization, none of which revealed any arrhythmias. Bubble test excluded patent foramen ovale. Lower limb vascular ultrasound excluded deep vein thrombosis in the lower limbs. Cranial MRI revealed no evidence of cerebral infarction (**Figures 3A, B**). CTA showed right vertebral artery stenosis, and no significant abnormalities were observed in the aortic arch (**Figure 3C**). The patient was diagnosed with a relapse of the ABP variant of GBS. He was treated with IVIg at 0.4 g/kg/day for 5 days, which was initiated on August 17.

He developed weakness in both upper limbs (4/5 MRC) on August 20, and D-dimer and fibrinogen degradation products were elevated (**Figure 2**, right). MRI revealed multiple cerebral infarctions in the bilateral parieto-occipital and left frontal lobes on August 22 (**Figures 1D–F**). LMWH was added to the treatment plan after cerebral infarction. Upon discharge, his swallowing function and muscle strength in both upper limbs (4/5 MRC in the right distal upper limb and 5/5 in the other limbs) partially recovered. After 3 months, the patient completely recovered, with an mRS score of 1.

## 3 Discussion

Herein, we report two cases of GBS in patients who developed multiple cerebral infarctions after IVIg therapy. We searched the Web of Science, Scopus, Embase, and PubMed up to March 2024 using medical subject headings of “Guillain–Barré syndrome,” “Miller Fisher syndrome,” “intravenous immunoglobulin,” and one of the terms: “ischemia,” “infarction,” or “stroke.” Finally, we identified ten articles related to GBS and cerebral infarction and gathered relevant data from these articles (8–17) (**Tables 1**, **2**).

**Table 1:** Demographics, clinical features, and outcomes of 10 GBS patients with cerebral infarction complications after IVIg.

Demographics	
n	10
Median age (mean), years	57.9 (29−84)
Male sex	40% (4/10)
Clinical features	
Prodromal infection	50% (5/10)
Cranial nerve paralysis	60% (6/10)
Limb weakness	80% (8/10)
Paresthesia	30% (3/10)
Loss of tendon reflexes	90% (9/10)
Ataxia	30% (3/100)
Risk factors	
Hypertension	20% (2/10)
Type 2 diabetes	10% (1/10)
SIADH	20% (2/10)
Coagulation dysfunction	10% (1/10)
Cerebral vascular stenosis or spasm	30% (3/10)
**Days from IVIg to the occurrence of stroke (median)**	4 (during treatment-25)
Affected areas of circulation	
Anterior	10% (1/10)
Posterior	70% (7/10)
Anterior and posterior	20% (2/10)
Distribution of lesions	
Single	30% (3/10)
Multiple	70% (7/10)
Outcome	
Good outcome (mRS 0−2)	50% (5/10)
Unfavorable outcome (mRS 3−5)	30% (3/10)
Death	20% (2/10)

GBS, Guillain–Barré syndrome; mRS, modified Rankin Scale; IVIg, intravenous immunoglobulin; SIADH, syndrome of inappropriate antidiuretic hormone secretion.

**Table 2:** Characteristics of 10 patients with GBS and cerebral infarction complications after IVIg.

Reference	Sex, Age (years)	Clinical features	Diagnosis	Days from IVIg to occurrence of stroke	Affected brain region	Numbers of lesions	Outcome (mRS)
Silbert et al. (1991) (8)	Male, 84	Dysarthria, dysphagia	GBS	1	Multiple areas of both cerebral hemispheres, the right parietal region	Multiple	Death
Turner, and Wills (2000) (9)	Female, 60	Cranial nerve paralysis, limb weakness, loss of deep tendon reflexes, ataxia	MFS	3	Bilateral occipital lobe	2	2
Velioğlu et al. (2001) (10)	Male, 55	Limb weakness, loss of deep tendon reflexes	GBS	2	Left parieto-occipital lobe	2	2
Byrne et al. (2002) (11)	Female, 70	Cranial nerve paralysis, limb weakness, paresthesia	GBS	8	Bilateral parietal and posterior temporal lobes	2	Death
Doss-Esper et al. (2005) (12)	Female, 66	Cranial nerve paralysis, limb weakness, loss of deep tendon reflexes	GBS	During treatment	Left splenium and occipital lobe	2	4
Saeed et al. (2010) (13)	Male, 62	Limb weakness, loss of deep tendon reflexes	GBS	5	Bilateral occipital lobe	2	2
Chang et al. (2014) (14)	Male, 44	Cranial nerve paralysis, limb weakness, paresthesia, ataxia	MFS	25	Left parieto-occipital lobe	1	2
Stetefeld et al. (2014) (15)	Female, 29	Cranial nerve paralysis, ataxia	MFS	During treatment	Left parieto-occipital lobe	1	2
Prateek et al. (2019) (16)	Female, 30	Limb weakness, loss of deep tendon reflexes	GBS	13	Left parieto-occipital lobe	1	4
Chun et al. (2021) (17)	Female, 79	Limb weakness, loss of deep tendon reflexes	GBS	3	Left anterior cerebral artery territory	multiple	4

GBS, Guillain–Barré syndrome; MFS, Miller Fisher syndrome; mRS, modified Rankin Scale.

Of the population, 60% were female, with an average age of 57.9 years (range, 29–84 years), including seven cases of GBS and three cases of MFS. More than half the patients presented with prodromal infections, cranial nerve paralysis, limb weakness, or loss of tendon reflexes. Some patients had a history of syndrome of inappropriate antidiuretic hormone secretion (SIADH), hypertension, and type 2 diabetes, and laboratory tests revealed cerebrovascular stenosis and coagulation disorders. The median time from IVIg administration to the occurrence of cerebral infarction was 4 days (the longest was up to 25 days). Symptoms of cerebral infarction included cortical blindness, aphasia, hemiplegia, and coma. Cerebral infarction mainly affected the posterior circulation, including the occipital and parietal lobes. Precisely 70% of patients had more than one cerebral infarction. The prognosis of 50% of patients was relatively good.

IVIg treatment has been more commonly utilized for managing various acute and chronic immune-related neurological disorders and is indicated as a dependable and efficient strategy (5). Unwanted responses to IVIg treatment usually are minor. Prior publications about the prevalence of undesired outcomes, such as muscle pain, migraine, high temperature, and asymptomatic laboratory changes, exhibit a broad spectrum from 11−81% (18). Significant issues, such as cerebrovascular accidents, kidney dysfunction, and hepatitis, have rarely been documented.

Cerebral infarction after receiving IVIg is uncommon and is believed to be connected to vascular risk factors. The risk of TEE increases with an increase in the number of cardiovascular risk factors (such as high blood pressure, heart disease, diabetes, and smoking) (19). The chances of encountering TEE within a fortnight of IVIg therapy were 10 times greater in the presence of ≥4 cardiovascular risk factors (20). The two patients we reported had a history of smoking, coronary heart disease, and vertebral artery stenosis, which is consistent with previous reports.

Although the mechanism by which IVIg causes stroke remains unclear, several explanations have been proposed. First, IVIg can lead to increased serum viscosity (21). Although this increase may not affect healthy individuals, patients with vascular risk factors and other pre-existing high serum viscosity conditions may have a greater risk of TEE. Previously, there have been reports of two cases of MFS combined with SIADH, where water intake was restricted while receiving IVIg therapy (9, 14). The occurrence of cerebral infarction in these two cases may have been due to increased plasma viscosity caused by inadequate blood volume. Second, IVIg treatments could potentially contain coagulation factor XI impurities, resulting in the synthesis of a substantial quantity of thrombin and subsequent vascular thrombosis (22). One patient showed intravascular platelet-fibrin-IgG thrombi in the infarcted regions and fibrin-IgG globules in viable areas during autopsy (11). We monitored the coagulation function of our two patients and found that the levels of D-dimer and fibrinogen degradation products were significantly elevated after IVIg treatment (**Figure 2**), suggesting vascular thrombosis and secondary fibrinolysis. Third, IVIg therapy may cause cerebrovascular spasms, leading to ischemia and possible thrombosis (23). It is worth mentioning that our first patient had a congenital bicuspid aortic valve anomaly with regurgitation, which may have caused local turbulence. Coupled with the effect of hypercoagulability, this could lead to the formation of microthrombi, resulting in multiple cerebral infarctions predominantly affecting the cortex.

Regarding the treatment approach, the two patients in this study did not undergo prophylactic anticoagulation during immunoglobulin therapy but were administered LWMH for anticoagulation after the onset of cerebral infarction. In our review of case reports, one case specifically reported the use of LWMH prophylactic anticoagulation at the start of IVIg, while the other nine cases did not mention it. Additionally, among the 10 patients diagnosed with cerebral infarction, one received antiplatelet therapy, one received LWMH anticoagulation therapy, one received oral anticoagulant therapy, and the treatment methods for cerebral infarction in the remaining seven patients were not reported. A retrospective study found that the incidence of thrombotic events was 1.96% (3/153) in patients who received anticoagulant prophylaxis based on individual thrombotic risk factors, compared to 1.1% (2/181) in patients who systematically received anticoagulant prophylaxis after IVIg infusion (24). There was no difference in the incidence of thrombotic events between the two groups, likely due to the low incidence of thrombotic events (24). Therefore, the effectiveness and safety of different thromboprophylaxis methods, including LWMH and antiplatelet drugs, should be analyzed in large-sample prospective cohort studies.

Our two patients showed significant recovery at the 6-month follow-up after cerebral infarction, similar to half of the patients reviewed whose prognoses were favorable. IVIg can decrease inappropriate activation of inflammation and the immune system and also protect the nervous system (25). IVIg can block activation of the complement system and infiltration of white blood cells, adjust the network of cytokines, and prevent neuronal cell death in cases of ischemic stroke (26). The favorable prognosis may be associated with the neuroprotective effects of immunoglobulin therapy, although the specific mechanisms require further research for confirmation.

## 4 Conclusion

Overall, patients should be administered a slower rate of infusion and smaller doses of IVIg, particularly in the presence of vascular risk factors. Patients should have careful fluid balance management and be closely monitored for potential strokes associated with thrombosis during IVIg therapy.

## Data availability statement

The original contributions presented in the study are included in the article. Further inquiries can be directed to the corresponding authors.

## Ethics statement

The studies involving humans were approved by Shanghai Changhai Hospital Ethics Committee. The studies were conducted in accordance with the local legislation and institutional requirements. The participants provided their written informed consent to participate in this study. Written informed consent was obtained from the individual(s) for the publication of any potentially identifiable images or data included in this article.

## Author contributions

WW: Conceptualization, Investigation, Writing – original draft, Writing – review & editing. CF: Conceptualization, Investigation, Writing – original draft, Writing – review & editing. YL: Data curation, Writing – original draft. YT: Data curation, Writing – review & editing. XB: Funding acquisition, Supervision, Writing – review & editing. XH: Funding acquisition, Supervision, Writing – review & editing.
